# Supplementary material for: Disaggregation of Green Space Access, Walkability, and Behavioral Risk Factor Data for Precise Estimation of Local Population Characteristics
Source: Int J Environ Res Public Health. 2024 Jun 14;21(6):771. doi: 10.3390/ijerph21060771 (PMC11203488; doi:10.3390/ijerph21060771)
Supplement: Supplementary file 1 [file ijerph-21-00771-s001.zip › ijerph-2847504-supplementary.pdf]

Appendix A

Supplementary Table S1: Summary of the *Allegheny County – Greenways* dataset by "Type". Include = "Yes" for those Types of green spaces deemed usable for recreation by the public.

Type	Count	Sum area	Median patch size	Include
Allegheny Land Trust GREENPRINT	7583	1655468606	9441.4957	No
Sensitive Slope Areas	42726	1509670924	2863.133292	No
Rivers & Streams, Wetlands, Forested Floodplain	1455	969047763	84160.01337	No
Regional Parks	81	627673655	237215.5256	Yes
Golf Courses	51	315163245	5974481.743	No
Municipal Parks	755	279479351	115420.4671	Yes
Community Parks	129	251983197	650119.6355	Yes
Land Trust Property	74	89893406.8	202091.1484	Yes
Agricultural Easements	9	42197876.6	3432002.199	No
City of Pittsburgh Greenways	42	19635520.1	114790.5359	Yes
Park Node	127	14535370.9	46238.90449	Yes
Trails	2	13119499.3	6559749.652	Yes
Bike Lane/Share the Road	1	522.133278	522.133278	No

Supplementary Table S2. Combined ranking of zip codes in the Allegheny County based on BMI, Ever-smoker and Moderate activity. (Ni represents the population size of the zip code, and ni the corresponding sample size. NA implies that sample information is not available for the zip code.)

Zip code	BMI			Ever-smoker			Moderate activity			Median Rank
	Rank	Ni	ni	Rank	Ni	ni	Rank	Ni	ni	
15003	60	3209	17	39	403	2	42	2512	14	42
15005	54	4784	31	53	790	3	58	4048	25	54
15006	49	5758	27	42	3250	18	13	4902	22	42
15007	74	11097	65	34	4784	31	98	8582	51	74
15014	75	246	2	71	5869	28	65	246	2	71
15015	90	1062	4	92	11097	65	63	1062	4	90
15017	99	876	7	72	1062	4	34	333	2	72
15018	29	2725	14	33	876	7	56	738	6	33
15019	20	7861	41	24	2725	14	77	2305	11	24
15020	33	8644	51	37	8000	42	NA	NA	NA	35
15024	62	5099	25	112	8829	52	106	7032	36	106
15025	23	3286	15	46	5099	25	66	8319	49	46
15026	108	595	4	60	758	4	4	3693	19	60
15028	25	13112	54	45	557	3	NA	NA	NA	35
15030	12	2703	9	58	483	2	41	758	4	41
15031	59	3329	13	55	277	2	7	557	3	55
15034	14	656	5	23	3286	15	20	483	2	20

15035	6	12183	56	5	595	4	105	2935	13	6
15037	78	10395	45	88	13491	56	36	484	3	78
15042	37	8251	52	61	2790	10	75	11560	48	61
15044	65	12938	81	96	3329	13	14	2790	10	65
15045	32	15935	104	11	656	5	49	2949	11	32
15046	48	6648	36	102	12529	58	3	656	5	48
15049	43	18762	100	108	343	2	103	11438	53	103
15051	64	2596	16	111	10395	45	1	9717	40	64
15056	NA	NA	NA	63	8798	55	94	7423	46	79
15057	40	2968	17	97	13211	83	101	12199	76	97
15063	10	7813	46	3	16845	112	5	15005	98	5
15065	50	8963	51	8	6943	38	99	6485	35	50
15068	103	12105	64	51	19395	105	83	17684	93	83
15071	4	3180	18	17	2596	16	6	1757	9	6
15075	8	5712	27	13	2968	17	109	2968	17	13
15076	110	10676	49	113	8102	48	68	7212	42	110
15082	77	17407	104	82	9464	55	55	6899	43	77
15083	46	11307	55	47	12933	69	89	12107	63	47
15084	16	3336	18	6	3180	18	90	2962	17	16
15085	70	22356	105	57	5712	27	52	5277	24	57
15086	92	15036	68	50	10864	50	39	7498	33	50
15088	NA	NA	NA	26	18253	108	78	13233	74	52
15089	55	1790	13	41	11776	58	74	10401	50	55
15090	104	3143	15	110	3336	18	104	2735	14	104
15101	47	198	2	70	23287	110	85	19899	94	70
15102	67	11236	54	104	15329	70	67	13037	54	67
15104	15	2274	9	12	2029	14	97	1917	13	15
15106	97	7089	38	80	3143	15	60	2639	12	80
15108	69	18380	116	105	317	3	46	317	3	69
15110	72	10032	72	31	11395	56	84	9642	48	72
15112	35	5133	23	2	2274	9	15	2164	8	15
15116	63	7448	46	67	7503	40	8	5784	33	63
15120	7	7394	42	29	19249	122	22	16705	105	22
15122	30	7846	40	30	10819	79	73	9222	68	30
15126	9	5178	30	4	5412	24	95	4239	17	9
15129	18	8110	51	25	7625	48	11	7242	44	18
15131	73	39537	235	14	7549	43	107	5648	34	73
15132	11	7415	34	44	8026	42	88	7356	37	44
15133	79	14530	100	9	5538	32	80	4626	26	79
15135	98	7277	39	52	8340	53	2	7806	48	52
15136	36	21257	115	56	40601	238	53	36185	204	53
15137	22	13584	67	28	8128	39	50	6054	30	28
15139	96	19412	112	103	14830	102	21	13052	87	96

15140	2	12007	76	59	7277	39	81	4954	29	59
15142	102	9999	67	106	21865	119	38	17926	92	102
15143	93	4737	25	98	13989	68	51	12864	62	93
15144	3	12825	61	109	20727	121	110	16374	94	109
15145	34	14894	88	68	12096	77	69	10444	64	68
15146	39	28124	136	65	10355	71	87	8050	54	65
15147	31	12568	116	22	4848	26	54	3578	20	31
15148	1	8617	48	19	13151	63	10	12408	58	10
15201	66	58099	383	7	15523	93	79	11926	72	66
15202	19	5266	28	75	28827	141	9	24567	109	19
15203	101	4683	19	73	12920	121	86	10261	97	86
15204	13	9887	56	21	8848	50	32	8438	47	21
15205	82	9690	45	18	59428	394	37	50028	321	37
15206	68	21144	100	81	5266	28	35	4434	23	68
15207	42	10904	62	32	4794	20	76	3766	17	42
15208	52	10391	51	77	10026	57	102	7894	49	77
15209	87	10935	45	62	661	5	26	8373	37	62
15210	26	1726	11	10	10045	47	70	19063	89	26
15211	57	7934	46	78	21209	101	29	9675	56	57
15212	24	39439	261	20	10791	61	91	9251	47	24
15213	91	13656	83	79	10721	53	93	10135	38	91
15214	27	22176	132	27	10935	45	31	1633	10	27
15215	81	7527	44	89	1791	12	108	6389	37	89
15216	107	9428	52	38	8153	47	61	34473	226	61
15217	88	8801	54	95	40658	268	30	11920	76	88
15218	84	5201	31	90	14572	89	16	19900	117	84
15219	21	791	4	15	22434	134	96	6439	37	21
15220	58	403	2	69	7457	43	48	8407	47	58
15221	53	89	1	49	9586	53	44	7288	45	49
15222	111	158	1	64	9064	56	71	5421	34	71
15223	95	790	3	94	5833	37	72	791	4	94
15224	56	208	2	16	89	1	82	403	2	56
15225	41	594	2	1	158	1	62	89	1	41
15226	28	692	1	83	208	2	59	158	1	59
15227	17	243	1	85	594	2	40	790	3	40
15228	109	178	1	99	692	1	24	208	2	99
15229	80	333	2	54	243	1	64	594	2	64
15232	86	331	1	101	246	2	57	692	1	86
15233	106	758	4	40	178	1	25	331	1	40
15234	45	445	2	86	333	2	100	277	2	86
15235	38	483	2	36	331	1	18	185	1	36
15236	61	185	1	43	185	1	92	331	1	61
15237	85	331	1	91	331	1	43	398	1	85

15238	51	398	1	107	398	1	33	331	1	51
15239	5	331	1	66	331	1	19	343	2	19
15241	94	343	2	93	190	1	45	190	1	93
15243	105	154	1	100	154	1	12	154	1	100
15321	76	661	5	76	177	1	23	368	2	76
15332	100	177	1	35	791	4	17	177	1	35
15642	71	342	1	74	342	1	47	342	1	71
15644	44	405	1	48	405	1	28	405	1	44
15668	83	990	1	84	990	1	NA	NA	NA	84
16059	89	89	1	87	89	1	27	89	1	87
